# Gelsemium Sempervirens

**Published:** 1891-05-23

**Authors:** 


### THERAPEUTICS.

### GELSEMIUM SEMPERVIRENS.

The rhizome and rootlets of the Gelsemium sempervirens, or Carolina jasmine contain the active principle gelsemine. The plant is one of the most beautiful tree creepers of Florida, having a yellow flower which is said to be poisonous. Gelseminic acid was first investigated by Wormley, more recently by Robbins, who concluded that it was identical with æsculin, the glucoside of Æsculus hippocastanum, the common horsechestnut. The drug owes its therapeutical properties, however, to the alkaloid gelsemine, the formula of which is stated to be $C_{12}\ H_{14}\ NO_2$.

The so-called physiological, but more correctly termed pathological, action of gelsemine has been observed in many cases of poisoning by gelsemium. Moderate doses give rise to injection of the conjunctivæ, pain in the eye-lids, and contracted pupils, with slight ptosis. A sense of not unpleasant languor, with muscular weakness, and a consequent difficulty in keeping the jaw closed are early symptoms of poisoning.

Larger doses affect particularly the motor tracts of the spinal cord at first, loss of voluntary movement with staggering, followed by motor paralysis, and finally tetanic convulsions resembling strychnine poisoning. Previous to the onset of tetanus fibrillary movements are observed in the muscles, and the tetanic convulsions differ from those produced by strychnine in occurring rhythmically and not being persistent, and there is marked implication of the respiratory centres. The tetanic convulsions are soon followed by persistent paralysis. The first nerve affected in gelsemium poisoning is the sixth at its termination, and thus, by paralysis of the external rectus muscle, internal strabismus and double vision is produced. This is soon followed by implication of the third with ptosis and dilated pupils. In the earlier stages the pupils are contracted. Dizziness, numbness, or anæsthesia, or a marked lowering of the temperature. It has a depressing action on the heart, and the pulse rate is diminished, probably by its paralysing action on the excito-motor ganglia of the heart. Its action on the respirations, which become shallow, laboured, and arrhythmical are likewise the result of implication of the respiratory centres, death resulting from failure of respiration. Berger, however, has stated that death results from paralysis of the vagus.

The therapeutical applications of gelsemium may be arranged under the following headings:

In spasmodic affections it has been employed on account of its paralytic action on the spinal centres. It has been recommended for tetanus. It has been administered in local spasmodic affections with success, such as blepharospasm, spasmodic torticollis, painters' cramp, and also in chorea. Dr. Read has reported a case of tetanus which was cured by forty-drop doses of tincture of gelsemium every two hours as long as the convulsions recurred, followed by twenty-drop doses every two hours for several days. Bartholow recommends the tincture of gelsemium in certain forms of convulsive or spasmodic cough, and in acute inflammations of the lungs and plura, he thinks it may do good by diminishing the activity of the respiratory functions.

Gelsemium is most frequently employed in *neuralgic affections*. It is in neuralgias of the fifth nerve that it is most certain in action, such as facial neuralgias, tic douloureux, and inferior dental neuralgia. But it has also proved successful in ovarian and intercostal neuralgia, and in myalgia. The dose should be about five or ten minims of the tincture repeated every hour till relief is obtained, but inasmuch as the strength of the tincture varies much, as well as individual susceptibility to the drug, it is safer to begin with doses of two to five minims, gradually increasing it. Early symptoms of poisoning should be carefully watched for, as several fatal cases have been reported from overdosage.

Gelsemium has been used in America as a febrifuge, although in such a role it has never found much favour. It is hardly likely to replace the very numerous, efficient, and safe antipyretics that have been introduced within the last five years, but it is useful in some of the febrile attacks of children.

As a hypnotic it is certainly worthy of consideration. In insomnia from exhaustion, or the result of excitemen, the dose must be small, half to two minims, or not exceeding five, and may be repeated at intervals of an hour. It probably acts as a gentle stimulant to the exhausted nerve centres.

